# Evaluation of Feedstuffs as a Potential Carrier of Avian Influenza Virus between Feed Mills and Poultry Farms

**DOI:** 10.3390/pathogens11070755

**Published:** 2022-07-02

**Authors:** Shahan Azeem, Yuko Sato, Baoqing Guo, Anna Wolc, Hanjun Kim, Hai Hoang, Mahesh Bhandari, Kathleen Mayo, Jian Yuan, Jihun Yoon, Phillip C. Gauger, Kyoung-Jin Yoon

**Affiliations:** 1 Department of Veterinary Microbiology and Preventive Medicine, College of Veterinary Medicine, Iowa State University, Ames, IA 50011, USA; sazeem@uvas.edu.pk (S.A.); hai.hoangthanh@hcmuaf.edu.vn (H.H.); vet.bhandari@gmail.com (M.B.); kmayo@insta-pro.com (K.M.); 2 Institute of Microbiology, Faculty of Veterinary Science, University of Veterinary and Animal Sciences, Lahore 54000, Pakistan; 3 Department of Veterinary Diagnostic and Production Animal Medicine, College of Veterinary Medicine, Iowa State University, Ames, IA 50011, USA; ysato@iastate.edu (Y.S.); bqguo@iastate.edu (B.G.); hkim16@iastate.edu (H.K.); leon19841@gmail.com (J.Y.); pcgauger@iastate.edu (P.C.G.); 4 Department of Animal Science, College of Agriculture and Life Sciences, Iowa State University, Ames, IA 50011, USA; awolc@iastate.edu; 5 Hy-Line International, Dallas Center, IA 50063, USA; 6 College of Engineering, Iowa State University, Ames, IA 50011, USA; jihunyoon1226@gmail.com

**Keywords:** avian influenza virus, poultry, feed, complete layer mash, real-time polymerase chain reaction, half-life

## Abstract

The present study was conducted to assess the potential vector role of feedstuffs for the area spreading of avian influenza virus (AIV). Firstly, feed samples were collected from commercial poultry facilities that experienced highly pathogenic avian influenza (H5N2) in 2014–2015 for AIV testing by a real-time RT–PCR specific for the viral matrix gene. Secondly, feed materials obtained from an AIV-negative farm were spiked with various concentrations of a low pathogenic AIV H5N2. Virus-spiked cell culture media were prepared in the same manner and used for comparison. The spiked feed and media samples were tested by a multiplex real-time RT–PCR ran in a quantitative manner, either immediately or after incubation at −20, 4, 22, and 37 °C for 24, 48, and 72 h. Some of the feedstuffs collected from the poultry facilities or feed mills were positive for AIV RNA but negative by the virus isolation (VI) test, while all the formaldehyde-treated feedstuffs were PCR-negative. In the spiked feeds, the AIV titer was 1–3 logs lower than that in the corresponding media, even when tested immediately after spiking, suggesting that feed might have a negative impact on the virus or PCR detection. The half-life of AIV RNA was shorter at a higher temperature. A significant decay in the viral RNA over time was noted at 37 °C (*p* < 0.05), suggesting that feedstuffs should be maintained in the cold chain when testing is desired. Furthermore, the thermal degradation of AIV suggests that the heat treatment of feeds could be an alternative to chemical treatment when contamination is suspected. Collectively, the study observations indicate that AIV survivability in feed is relatively low, thus rendering it a low risk.

## 1. Introduction

Influenza A virus (IAV) is an enveloped RNA virus, is a member of the *Orthomyxoviridae* family, and is known to infect humans, mammals, and birds [1]. The virus is subtyped based on the antigenic cross-reactivity and sequence identity of two surface glycoproteins, namely, hemagglutinin (HA) and neuraminidase (NA) [2]. Excluding the recently identified H17N10 and H18N11 in bats, 16 HA and 9 NA IAV subtypes have been identified in mammals and birds to date [3,4].

Migratory wild birds (MWBs), including ducks, geese, and swans, serve as a natural reservoir for IAV and are known to harbor all known subtypes of the virus and most subtype combinations thereof [2,5]. Influenza A viruses of avian origin, also known as avian influenza viruses (AIVs), including highly pathogenic AIV (HPAIV), can occasionally spill over from MWBs to poultry [6]. The emergence of HPAIV in MWBs, coupled with their extensive movement across the globe and the asymptomatic infection with some HPAIVs, place MWBs at the center of AIV ecology and in a position to pose a serious threat to poultry. This is exemplified by the fact that all recent epornitics of HPAI in poultry, including the H5N1 US outbreaks of 2020–2022 [7], H7N3 in South Carolina [8], and H5Nx, have been associated with MWBs, confirming that the MWBs pose a real risk of introducing AIVs to poultry [6,9,10].

The 2014–2015 HPAI epornitic had a devastating effect on the US poultry industry, particularly in the Midwest. The epornitic spread to 21 states and led to the loss of over 50 million poultry. In the state of Iowa alone, the total economic loss was estimated at USD 1.2 billion [11]. Nationwide, the losses are estimated to be USD 5 billion [12]. HPAIV H5N2, the causative AIV strain of the 2014–2015 epornitic, was reported to have its origin in migratory waterfowl [13,14]. However, an epidemiological investigation suggested that both the initial introduction of H5N2 AIV by migratory waterfowl and its spread among poultry facilities contributed to the epornitic [15]. Since most commercial poultry production takes place in controlled confinement, which limits migratory waterfowl–poultry interaction, it was speculated that further virus spread may have occurred through various vectors [15]. Furthermore, studies performed by the Southeastern Poultry Research Laboratory suggest that the median bird infectious dose of the 2014–2015 HPAIV H5N2 is 1000 times higher than other HPAI viruses. Thus, fomites, if involved, could carry a relatively large amount of the virus to play a role in its spread [16].

Research on the past avian influenza outbreaks in the US suggests that contaminated feed trucks can introduce the virus to new locations and help spread the virus among farms [17]. In the field, feedstuffs, especially those stored in open bins, can be contaminated by wild bird droppings that may contain AIVs [18]. Moreover, strict biosecurity compliance is sometimes hard to achieve in feed mills and feed trucks, as some companies outsource their services. Considering these circumstances, the present study was conducted to determine if feedstuffs can be contaminated with AIVs and serve as a potential carrier, becoming a source of the virus to commercial poultry operations.

## 2. Materials and Methods

### 2.1. Collection of Feedstuffs from Commercial Poultry Facilities

Commercial feed ingredients and a complete layer mash were collected from three US Midwestern barns and four feed mills with a confirmed 2015 HPAI outbreak. Sampling was made from each facility after all the birds were depopulated. Each facility represented approximately 0.25–1.5 million layers. A commercial layer mash instead of pellets was chosen for this virus study because the pelleting process is known to inactivate AIV and also because pelleting is not routinely used in the US layer industry [18].

The feed samples were collected according to a standard collection guideline, minimizing cross-contamination between the samples. Sampling from the interested parties included approximately two pounds of feed per affected poultry farm (feed trough/feeders, hoppers, and silos), as well as from the feed mills located in or close to the affected zones after each control point (raw ingredient, post-grinding or processing, post-formaldehyde treatment). Collaborators were asked to store feed samples in a Ziploc^®^ bag, wipe the exterior of the bag with 70% ethanol, place it in a secondary Ziploc^®^ bag, and ship it chilled to the Iowa State University Veterinary Diagnostic Laboratory (ISUVDL) for testing. At the ISUVDL, the feed samples were processed as described below and subjected to a polymerase chain reaction (PCR)-based assay and virus isolation (VI) on PCR-positive samples.

### 2.2. Preparation and Testing of Virus-Spiked Feeds

An AIV-negative layer mash (10% moisture) was obtained from the ISU poultry research farm. The layer mash was spiked with an LPAIV H5N2, A/Mallard/Delaware/A00060540/2007, to yield the final viral titer of 10^6^–10^0^ median embryo infective dose (EID_50_) per 1 g of feed. In order to spike the layer mash, a nebulizing device (LMA MAD Nasal Intranasal Mucosal Atomization Device (Teleflex, Morrisville, NC, USA) was used. After being spiked, the feed was vortexed for the thorough and uniform mixing of AIV with it. The spiked feed containing the concentrations of the virus mentioned above was prepared in a container sufficient for each temperature and time point. A 20-g aliquot of it was incubated at −20 °C, 4 °C, 22 °C, and 37 °C for 24, 48, and 72 h, respectively, and stored at −80 °C until tested by the PCR assay, as described below. A cell culture medium (DMEM) containing the final viral titer of 10^6^–10^0^ EID_50_/mL was also maintained under the same conditions. Each treatment was run in triplicate. A subset of each viral concentration was not incubated to serve as a “zero hour” (0 h) sample to compare the effect of storage time.

Virus isolation was attempted on spiked feed and media samples containing 10^6^–10^3^ EID_50_ of HPAIV H5N2 per 1 g or 1 mL, respectively, if they were positive by PCR after incubation at −20 and 22 °C for 0 and 72 h. Those concentrations were selected as those samples most likely contain the virus. The −20 °C temperature was selected since it would be the ideal condition for virus survival. Such temperature also represents the US Midwest winter. The 22 °C temperature represents room temperature. Likewise, a time of up to 72 h was selected to mimic a potential time elapse from collection in the field to testing in a diagnostic laboratory and its effect on the AIV testing outcomes.

### 2.3. Feed Sample Processing

Five grams of feed were mixed with twenty mL of phosphate-buffered saline (PBS), pH 7.2 and vortexed. The feed tubes were placed in a refrigerator (5 ± 3 °C) overnight and were subsequently centrifuged at 4200× *g* for 10 min for clarification. The resulting supernatants were saved and used in testing by PCR or VI.

### 2.4. Polymerase Chain Reaction-Based Assay

A commercial one-step real-time multiplex RT–PCR kit (VetMAX™-Gold AIV Detection Kit; Life Technologies, Austin, TX, USA) designed to target the matrix (M) and nucleoprotein genes of AIV was used to detect the presence of influenza A viral RNA, as per the protocol recommended by the manufacturer. The PCR reaction was set up in a 25 µL volume containing 12.5 µL of 2X multiplex RT–PCR buffer, 1.0 µL of nuclease-free water, 1.0 µL of influenza virus primer-probe mix, 2.5 µL of multiplex RT–PCR enzyme mix, and 8.0 µL of RNA template (i.e., extract) or controls. The “Xeno™ RNA Control” supplied with the kit was included as an internal positive control for RNA purity and to assess the presence of possible PCR inhibitors in the samples. The “Influenza Virus-Xeno™ RNA Control Mix” included in the kit was used as a positive amplification control (PAC) for the real-time RT–PCR components and to set the cycle threshold (Ct) for evaluating the test results. Nuclease-free water was used as a no-template control. Thermocycling was performed in a 7500 Fast PCR System (Applied Biosystems, Foster City, CA, USA) under the following conditions: reverse transcription at 48 °C for 10 min, reverse transcriptase inactivation/initial denaturation at 95 °C for 10 min, and 40 cycles of amplification and extension at 95 °C for 15 s and at 60 °C for 45 s.

The PCR data were analyzed as per the recommendations of the manufacturer using the “Manual Cycle Threshold (Ct)” setting and the default baseline cycle 3–15. The AB AIV master detector threshold was determined by multiplying the delta Rn of PAC at cycle 40 by 0.05. The amplification plots were reviewed to ensure that the positive controls crossed the threshold but the negative controls did not. The AIV RNA and Xeno™ RNA controls were detected by using FAM™ and VIC™ dyes, respectively. For the experimentally virus-spiked samples, samples with Ct < 38 were recorded as positive for influenza A viral RNA, as per the manufacturer’s recommendation. However, feed samples collected from commercial facilities were called “Presumptive positive” if Ct values were <40, as per the USDA guideline, since only the USDA was authorized to declare the positive status of poultry and feed mill facilities.

### 2.5. Virus Isolation Assay

Virus isolation was carried out in 9- to 11-day old, specific-pathogen-free embryonated chicken eggs using the allantoic route inoculation method. Briefly, an antibiotic-antimycotic solution was added to each processed feed supernatant [19]. The solution contained penicillin G (2000 IU/mL), streptomycin sulfate (0.2 mg/mL), gentamicin sulfate (0.25 mg/mL), and amphotericin B (500 IU/mL) and was adjusted at pH 7.2 [20]. The samples were vortexed after the addition of the antibiotic-antimycotic solution and incubated at ambient temperature for approximately 2 h to inactivate possible contaminants [21]. After incubation, each feed sample (0.1–0.2 mL) was inoculated in two to three eggs. The inoculated eggs were maintained at 37 °C in a humidified egg incubator. The eggs were monitored daily by candling for embryo death. Eggs with an embryo death within 24 h post-inoculation were discarded, except for eggs inoculated with the samples from commercial poultry facilities. Allantoic fluids were harvested from the eggs either on the day of the detection of the embryo death or, if no embryo death was noted, at the end of a 5-day incubation period after inoculation. Allantoic fluids were centrifuged for 15 m at 1500× *g*. Virus growth in embryonated eggs was confirmed by a hemagglutination assay, followed by a real-time RT–PCR for the M gene of AIV on HA-positive egg fluids for further confirmation [22]. Primers and probe information for this PCR can be found elsewhere [23]. Thermocycling was performed in a 7500 Fast PCR System (Applied Biosystems, Foster City, CA, USA) under the following conditions: reverse transcription at 45 °C for 10 min, reverse transcriptase inactivation/initial denaturation at 95 °C for 10 min, and 45 cycles of amplification and extension (95 °C for 15 s and 60 °C for 45 s) [23]. One blind passage was made on each sample before the samples were considered negative for AIV.

### 2.6. Data Analysis

The estimation of the half-life of viral RNA in the feed and media samples Log_10_-transformed EID_50_ values was analyzed with a linear model using the “lm” function in the R software (version 3.3.3) [24]. The EID_50_ values were increased by one to enable log transformation for negative samples. A covariate of the log_10_-transformed initial viral dose was fitted to be adjusted for the starting level of the virus load and the fixed effect of the sample type to account for differences in the means between the feed and media. A covariate of time (0, 24, 48, 72 h) was fitted to estimate the decline rate of the virus. The interaction with the sample types and the decline rates of the viral titer was estimated to determine the differences between the feed and media [25]. The half-life (*t*_1/2_) of H5N2 LPAIV was determined separately in both the feed and media [26] at each temperature by fitting a simple linear regression model, accounting for the initial viral concentration [25,26]. Once the regression equation was constructed, the estimate of the slope and standard error were used to obtain a point estimate of the half-life and its confidence intervals, as previously reported [26]. If the slope was not significantly different from zero, the upper bound of the half-life confidence interval was incalculable and interpreted as infinity. The estimates of AIV *t*_1/2_ in the feed was compared to the media, which served as ideal storage material for assessing the survival of the virus in the feed. If more decay was noted in the feed or media, this was interpreted as the poor survival of the virus.

### 2.7. Estimation of the Limit of Detection of RRT–PCR in Feed Samples

The limit of detection (LOD) of the real-time multiplex RT–PCR was calculated by testing the spiked feed and media samples (10^6^ through 10^0^) in triplicate. The lowest concentration detected by the PCR in all three reactions (i.e., 100% detection) was recoded as LOD.

## 3. Results

### 3.1. Evaluation of Feed Samples from Commercial Operations

A total of 30 feed samples were submitted to ISUVDL, representing 14 different sample types, with shell corn and complete mash being the most common types. These samples represented seven poultry facilities and attached feed mills.

Twenty-one feed samples or feed ingredients representing two facilities were presumptively positive for AIV with high Ct values according to real-time multiplex RT–PCR. The PCR-positive samples represented nine barns in Facility #1 and four barns and an attached feed mill in Facility #2 (Table 1). In contrast, the formaldehyde-treated samples were all negative for AIV by the PCR.

Most of the positive feed samples showed relatively high Ct values (37.9–39.7); however, the samples collected from inside the feed troughs confirmed that virus-positive barns had lower Ct values (30.3–32.3). The only samples from the feed mill that tested positive were the eggshells and meat and bone meal. While the eggshells were sourced from flocks within the affected facility (i.e., internal contamination), the presumptive positive meat and bone meals were from an external source with relatively high Ct values close to 38. None of the PCR-positive samples yielded a viable virus on VI attempts.

### 3.2. Decay of Viral RNA in Feeds Spiked with LPAIV

**Figure 1:**
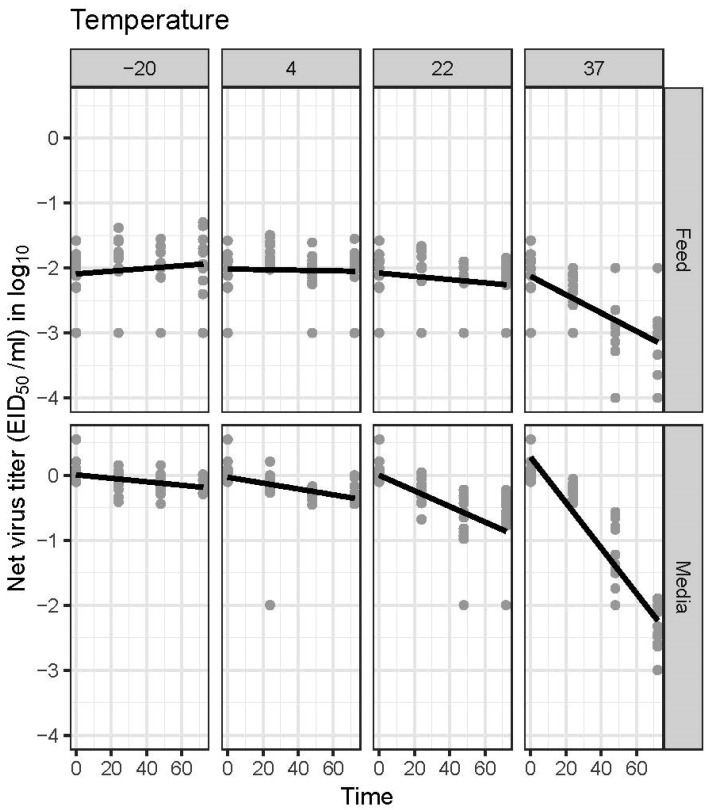
Changes in AIV RNA concentration in the feed and cell culture media (Dulbecco’s modified Eagle media) over time when incubated at various temperatures, as measured by a real-time RT–PCR. The net virus titer (EID_50_/ml equivalent) was calculated by subtracting the virus titer of spiked material from the estimated virus titer of sample after treatment.

The estimates of the AIV RNA half-life (h) are summarized in Table 2. The half-life of AIV RNA was shorter at a higher temperature than at lower temperatures and was significantly shorter in the media than in the feed. The shortest half-life estimate of 19.87 h was observed when the virus was stored at 37 °C in the media; at the same temperature, the half-life of the virus in the feed was 29.42 h longer compared to that in the media. Statistically, there was a substantial difference in the half-life of AIV between the feed and media when the spiked samples were incubated at 37 °C. This steep decline of AIV RNA at 37 °C in the media compared to the virus in the feed is illustrated in Figure 1. At 22 °C, a relatively longer half-life estimate or a slower rate of the decline of the virus was observed in the media, and at the same temperature, the decline rate in the feed was not significantly different from zero, thus leading to no meaningful half-life estimates. Lower temperatures (4 °C and −20 °C) maintained viral RNA that was stable in both the feed and media, with a decline rate not significantly different from zero, thus leading to no meaningful half-life estimates.

### 3.3. Analytic Performance of Commercial Real-Time Multiplex RT–PCR to Detect AIV RNA

The limits of detection (LOD) of the real-time multiplex RT–PCR for 100% detection of the AIV in the feed and media samples were 10^4^ EID_50_/g and 10^2^ EID_50_/mL, respectively, under the same experimental conditions, when the samples were tested immediately after spiking (Table 3). Surprisingly, the estimated AIV titer in the feed was 1 to 3 log10 lower than the corresponding media, even when tested immediately after spiking (Table 4). The LODs of the real-time multiplex RT–PCR for the virus in the feed samples were similar to that for the 0 h samples—10^3–4^ EID_50_/g, regardless of incubation temperatures—except for the feed stored at 37 °C for 48 and 72 h, which was 10^5^ EID_50_/g. The LOD of the PCR in the media was 10^2–3^ EID_50_/mL, regardless of incubation temperatures, except for the media stored at 37 °C for 72 h, when it was 10^4^ EID_50_/mL (Table 3). These data indicate a temperature effect on the virus, as was apparent on the LOD of the PCR.

### 3.4. VI Attempts on PCR-Positive Spiked Feeds and Media

No virus was isolated from any of the PCR-positive spiked feed samples regardless of whether it was tested immediately after preparation or after incubation at 22 °C and −20 °C for 72 h. In contrast, the viable virus was recovered from most of the corresponding media samples incubated at the same conditions for the same time (Table 5).

## 4. Discussion

The present study investigated contaminated feed as a potential carrier of AIV. Some feedstuffs collected from commercial layer facilities that experienced HPAI tested positive for AIV RNA but were negative by VI. Even with feeds experimentally spiked with LPAIV, a significant decay of viral RNA over time, as estimated by PCR, was observed when the spiked feeds were kept at 37 °C. The VI on a subset of PCR-positive spiked feeds did not yield a viable virus either. These observations suggest that the stability of the AIV in feedstuffs is not optimal, due to either the presence of inhibitory substances or the low moisture content of the layer mash feed. Due to the equivocal testing outcome (PCR versus VI), bioassays may have been a better choice for confirmation. Nevertheless, contamination with the virus should not be disregarded entirely, as viral RNA was detected in the feedstuffs, including the bone and meat meals from an external source.

All of the formaldehyde-treated feedstuffs were negative for AIV RNA, which agrees with a previous study demonstrating that formaldehyde treatment reduced the HPAIV level by 10^2^ to 10^8^ EID_50_/g of contaminated mash feed [18]. While AIV RNA could persist in spiked feeds for an extended period of time when stored at a low temperature, both the virus and viral RNA decayed quickly when the spiked feeds were stored at 37 °C. A previous study by other investigators has also shown the poor survivability of AIV in feeds [18]. These observations present a twofold implication for the poultry industry. Firstly, heat treatment prior to feeding could be a critical control point if contamination is an issue, providing an alternative to chemical treatment on certain feed ingredients. The present study indicated that both the feed and media required higher titers of the spiked virus to be positive on the PCR when kept at a higher temperature, suggesting a negative effect of the temperature on virus survival. Other investigators have also reported adverse effects of higher temperatures on the survival of AIV in various sample matrices and two swine RNA viruses (Senecavirus A and porcine reproductive and respiratory syndrome virus) in feeds [27,28,29,30]. Secondly, when testing is desired on feedstuffs, improper sample handling and storage may adversely affect the survivability of the virus and/or the stability of the viral genomic material in feeds, leading to false negatives. Therefore, feed samples should be kept chilled when they are shipped to a diagnostic laboratory for testing.

The mean bird infectious dose (BID_50_) of turkey-origin HPAIV H5N2 has been shown to be around 10^2.8^ to 10^4.7^ EID_50_/mL (median = 10^3.9^) [31]. The likelihood of a complete layer mash acquiring such a titer of virus from accidental contamination would be low, if not negligible, based on the observations from the present study. The current study showed that the amount of viral RNA in feedstuff from AIV-positive poultry facilities, when present, was very low, as the Ct values of the samples were close to the cut-off. The virus titer declined in the feed quickly, even after being spiked with a high titer of the virus under experimental conditions.

Taken together, feedstuffs appear to pose a low risk for AIV spreading, as suggested by others previously [18,32]. Nevertheless, appropriate biosecurity measures should be in place for screening feedstuffs of an external source and for on-farm feed management for safety in order to minimize the risk of AIV introduction via feedstuffs, because feed ingredients for pigs have been demonstrated as a vector for some swine viral pathogens [33,34,35,36].

## Figures and Tables

**Table 1:** Detection of avian influenza virus RNA in feedstuffs from commercial poultry barns and associated feed mills after an avian influenza outbreak. Sampling was done after all birds were depopulated.

	Sample Origin—Type	PCR Result (Ct/Interpretation)	Virus Isolation
Facility #1	Barn 1—Feed Trough 1	36.9/Presumptive positive	Negative
	Barn 2—Feed Trough 1	36.6/Presumptive positive	Negative
	Barn 2—Feed Trough 2	35.7/Presumptive positive	Negative
	Barn 3—Feed Trough 1	36.2/Presumptive positive	Negative
	Barn 3—Feed Trough 2	34.2/Presumptive positive	Negative
	Barn 4—Feed Trough 1	34.5/Presumptive positive	Negative
	Barn 4—Feed Trough 2	35.7/Presumptive positive	Negative
	Barn 5—Feed Trough 1	32.6/Presumptive positive	Negative
	Barn 5—Feed Trough 2	36.0/Presumptive positive	Negative
	Barn 6—Feed Trough 1	38.3/Presumptive positive	Negative
	Barn 7—Feed Trough 1	35.2/Presumptive positive	Negative
	Barn 8—Feed Trough 2	37.5/Presumptive positive	Negative
	Barn 9—Feed Trough 1	39.3/Presumptive positive	Negative
Facility #2	Barn 5—Feed Trough 3	32.2/Presumptive positive	Negative
	Barn 10—Feed Trough 4	32.3/Presumptive positive	Negative
	Barn 10—Feed Corners	30.5/Presumptive positive	Negative
	Barn 20—Feed Trough 2	31.1/Presumptive positive	Negative
	Barn 22—Feed Trough 1	30.3/Presumptive positive	Negative
	Feed mill, Egg Shells (internally sourced) ^A^	39.7/Presumptive positive	Negative
	Feed mill, Egg Shells (internally sourced)	38.1/Presumptive positive	Negative
	Feed mill, Meat and bone meal (externally sourced) ^B^	37.9/Presumptive positive	Negative

^A^ Internally sourced: ingredients came from company’s own flocks. ^B^ Externally sourced: ingredients came from an external entity.

**Table 2:** Half-life (h) of H5N2 low pathogenic avian influenza virus RNA in the feed and media. Dulbecco’s modified Eagle’s media at various temperatures.

	37 °C	22 °C	4 °C	−20 °C
Media	19.87 ^A^ (15.61, 27.36) ^B^	57.84 (31.16, 401.65)	∞ ^C^	∞
Feed	49.29 (30.36, 130.91)	∞	∞	∞

^A^ Half-life of AIV RNA was determined by a real-time reverse transcription–polymerase chain reaction (real-time RT–PCR). The feed or media was spiked with various concentrations (10^2^ to 10^6^ EID_50_/mL) of AIV and tested for virus titer by RRT–PCR immediately and every 24 h while incubating at various temperatures for 72 h. ^B^ 95% Confidence interval of the estimated half-life. ^C^ The slope was not significantly different from zero; hence, the upper bound of the confidence interval is incalculable and is interpreted as infinity.

**Table 3:** Limit of detection (AIV titer corresponding gray shadow) of real-time RT–PCR for H5N2 low pathogenic avian influenza virus spiked in the feed and media after 24, 48, and 72 h at various temperatures.

Temperature (°C)	Matrix	AIV Titer (EID_50_/mL or EID_50_/g) Expected in Each Virus-Spiked Sample													
		0 h							24 h						
		10^6^	10^5^	10^4^	10^3^	10^2^	10^1^	10^0^	10^6^	10^5^	10^4^	10^3^	10^2^	10^1^	10^0^
−20	Media	3/3 ^A^	3/3	3/3	3/3	3/3	1/3	0/3	3/3	3/3	3/3	3/3	3/3	0/3	0/3
4									3/3	3/3	3/3	3/3	2/3	0/3	0/3
22									3/3	3/3	3/3	3/3	3/3	0/3	0/3
37									3/3	3/3	3/3	3/3	3/3	0/3	0/3
−20	Feed	3/3	3/3	3/3	1/3	0/3	0/3	0/3	3/3	3/3	3/3	1/3	0/3	0/3	0/3
4									3/3	3/3	3/3	3/3	0/3	0/3	0/3
22									3/3	3/3	3/3	1/3	0/3	0/3	0/3
37									3/3	3/3	3/3	0/3	0/3	0/3	0/3
**Temperature (°C)**	**Matrix**	**AIV Titer (EID_50_/mL or EID_50_/) Expected in Each Virus-Spiked Sample**													
		**48 h**							**72 h**						
		**10^6^**	**10^5^**	**10^4^**	**10^3^**	**10^2^**	**10^1^**	**10^0^**	**10^6^**	**10^5^**	**10^4^**	**10^3^**	**10^2^**	**10^1^**	**10^0^**
−20	Media	3/3	3/3	3/3	3/3	3/3	1/3	0/3	3/3	3/3	3/3	3/3	2/2 ^B^	0/3	0/3
4		3/3	3/3	3/3	3/3	3/3	0/3	0/3	3/3	3/3	3/3	3/3	3/3	1/3	0/3
22		3/3	3/3	3/3	3/3	1/3	0/3	0/3	3/3	3/3	3/3	3/3	1/3	0/3	0/3
37		3/3	3/3	3/3	3/3	0/3	0/3	0/3	3/3	3/3	3/3	0/3	0/3	0/3	0/3
−20	Feed	3/3	3/3	3/3	2/3	0/3	0/3	0/3	3/3	3/3	3/3	1/3	0/3	0/3	0/3
4		3/3	3/3	3/3	1/3	0/3	0/3	0/3	3/3	3/3	3/3	1/3	0/3	0/3	0/3
22		3/3	3/3	3/3	0/3	0/3	0/3	0/3	3/3	3/3	3/3	0/3	0/3	0/3	0/3
37		3/3	3/3	0/3	0/3	0/3	0/3	0/3	3/3	3/3	0/3	0/3	0/3	0/3	0/3

^A^ Number of positive samples/Number of tested samples. ^B^ Result of one of the three replicate samples was not reported.

**Table 4:** PCR-based estimates of the virus titer (EID_50_ per 1 mL or 1 g) in the media and feed immediately after being spiked with an H5N2 low pathogenic avian influenza virus at various concentrations (10^0^ to 10^6^ EID_50_/mL).

Matrix	AIV Titer (EID_50_/h or EID_50_/g) Expected in Each Virus-Spiked Sample						
	10^6^	10^5^	10^4^	10^3^	10^2^	10^1^	10^0^
Media	1.0 × 10^6^ *	9.4 × 10^4^	8.6 × 10^3^	1.1 × 10^3^	1.9 × 10^2^	3.6 × 10^1^	Neg
Feed	7.9 × 10^3^	7.8 × 10^2^	1.3 × 10^2^	2.6 × 10^1^	Neg	Neg	Neg

* Virus titer in the sample estimated by a quantitative real-time RT–PCR.

**Table 5:** Presence or absence of infectious AIV in the feed and media spiked with various virus titers (10^3^ to 10^6^ EID_50_ per 1 mL or gram) immediately after spiking (0 h) or incubation at −20 °C and 22 °C for 72 h.

Temperature (°C)	Matrix	0 h				72 h			
		10^6^	10^5^	10^4^	10^3^	10^6^	10^5^	10^4^	10^3^
−20	Media	Pos (3/3)	Pos (3/3)	Pos (3/3)	Pos (3/3)	Pos (3/3)	Pos (3/3)	Pos (3/3)	Pos (3/3)
22						Pos (3/3)	Pos (3/3)	Pos (1/3)	Neg (0/3)
−20	Feed	Neg (0/3)	Neg (0/3)	Neg (0/3)	Neg (0/3)	Neg (0/3)	Neg (0/3)	Neg (0/3)	Neg (0/3)
22						Neg (0/3)	Neg (0/3)	Neg (0/3)	Neg (0/3)

## Data Availability

Data is contained within the article.

## Abbreviations

AIV = avian influenza virus; Ct = cycle threshold; DMEM = Dulbecco’s modified Eagle media; EID_50_ = median embryo infective dose; HA = hemagglutinin; HPAIV = highly pathogenic avian influenza virus; ISU = Iowa State University; ISUVDL = Iowa State University Veterinary Diagnostic Laboratory; LOD = limit of detection; LPAIV = low pathogenic AIV; NA = neuraminidase; PAC = positive amplification control; PBS = phosphate-buffered saline; PI = post-inoculation; RT–PCR = reverse transcription–polymerase chain reaction; RNA = ribonucleic acid; USDA = United States Department of Agriculture; VI = virus isolation
